# DNA in extracellular vesicles: biological and clinical aspects

**DOI:** 10.1002/1878-0261.12777

**Published:** 2020-08-19

**Authors:** Julia Elzanowska, Christine Semira, Bruno Costa‐Silva

**Affiliations:** ^1^ Champalimaud Research Champalimaud Centre for the Unknown Lisbon Portugal

**Keywords:** cancer, cell‐free DNA, EV‐DNA, extracellular vesicles, infection, liquid biopsies

## Abstract

The study of extracellular vesicles (EVs), especially in the liquid biopsy field, has rapidly evolved in recent years. However, most EV studies have focused on RNA or protein content and DNA in EVs (EV‐DNA) has largely been unnoticed. In this review, we compile current evidence regarding EV‐DNA and provide an extensive discussion on EV‐DNA biology. We look into EV‐DNA biogenesis and mechanisms of DNA loading into EVs, as well as describe the particularly significant function of DNA‐carrying EVs in the maintenance of cellular homeostasis, intracellular communication, and immune response modulation. We also examine the current role of EV‐DNA in the clinical setting, specifically in cancer, infections, pregnancy, and prenatal diagnosis.

## Graphical abstract

The presence of DNA in EVs, including exosomes and microvesicles, remains contentious as this field of study is still evolving. This review explores what is known about the biogenesis of EV‐DNA and the possible biological roles of DNA packaging into EVs. Moreover, we also discuss the potential clinical applications of EV‐DNA and how those compare to cell‐free DNA.

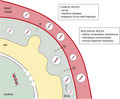

**Abbreviations**
BBB: blood–brain barrier
cfDNA: cell‐free DNA
cGAS: cyclic GMP‐AMP synthase
ctDNA: circulating tumor DNA
ds: double‐stranded
EGFR: epidermal growth factor receptor
EV‐DNA: DNA in extracellular vesicles
EV‐mtDNA: mtDNA in extracellular vesicles
EVs: extracellular vesicles
gDNA: genomic DNA
HBV: hepatitis B virus
HPV: human papillomavirus
IFN1: interferon type 1
ILVs: intraluminal vesicles
MAFs: mean frequencies
MN: micronuclei
mtDNA: mitochondrial DNA
MVBs: multivesicular bodies
NGS: next‐generation sequencing
NSCLC: non‐small‐cell lung cancer
PCR: polymerase chain reaction
PDAC: pancreatic ductal adenocarcinoma
ss: single‐stranded
STING: stimulator of interferon genes

## 1 Introduction

The study of extracellular vesicles (EVs) has come a long way since Chargaff and West [1] first reported the presence of platelet‐derived procoagulant particles after high‐speed centrifugation of human plasma in 1946. In the following decades, vesicles have been found to be shed from various tissues such as cartilage, epithelium, and even tumor cells, although the function of these vesicles remained unconfirmed [2, 3, 4]. It was only in the 1990s that EVs were identified to play a role in cell‐to‐cell communication, which sparked a growing interest in the field that persists until the present [5].

Currently, EVs are formally defined as lipid membrane‐bound vesicles secreted by cells into the extracellular space which are differentiated based upon their biogenesis, release pathways, size, content, and function [6]. The current consensus is that EV cargo is extremely heterogeneous—a varied combination of proteins, lipids, and nucleic acids and sugars [7, 8]. It has been recognized that EVs, with their corresponding content, function as molecular messengers, triggering intracellular signaling pathways [8]. In addition, EVs are easily accessible in body fluids and have emerged as a promising source of biomarkers of ongoing pathophysiological processes such as immune response, cell proliferation, or tumor metastasis [8].

While EV protein and mRNA have been extensively investigated, the functions and clinical value of DNA in EVs (EV‐DNA) have been less explored and still remain to be elucidated. To date, there are only a few studies regarding EV‐DNA, with most of it lumped under the umbrella of cell‐free DNA (cfDNA), although EV‐DNA represents a distinctive entity and adds another layer of complexity to EV research [9, 10]. In this article, we discuss the biology and possible mechanisms of EV‐DNA biogenesis and summarize existing studies on its function in human health and disease. We also compile the existing evidence on its potential clinical utility and identify the advantages of EV‐DNA over other biomaterials in liquid biopsies.

## 2 The presence and origins of cell‐free DNA in the circulation

The existence of cfDNA molecules in the human circulatory system was documented for the first time in the 1940s by Mandel and Metais [11]. From the 1960s onward, cfDNA started gaining clinical interest, as its elevated levels were detected in plasma and serum of patients suffering from diseases like rheumatoid arthritis and other autoimmune disorders [12, 13]. Around 10 years later, Leon *et al*. [14] documented for the first time the association between cancer and cfDNA, showing that cancer patients had increased cfDNA serum levels when compared to healthy subjects. This relationship was further confirmed by other groups [15].

Different subtypes of cfDNA have been characterized in the circulation, including double‐stranded (ds) and single‐stranded (ss) fragments, mitochondrial DNA (mtDNA), extrachromosomal circular DNA, as well as viral and bacterial DNA [16, 17]. In humans, cfDNA originates from all cells and it can be present in the circulation in different forms, mainly in macromolecular complexes (linked with proteins, lipids, or other nucleic acids) or associated with extracellular vesicles. This largely depends on the way the DNA is released from the cell, and a variety of passive and active DNA release mechanisms have been described in the literature. These include mainly apoptosis, necrosis, and active cellular secretion, as well as other pathways comprising neutrophil extracellular trap release, phagocytosis, and oncosis [18, 19, 20].

The characteristics of cfDNA, including concentration and size, vary considerably depending on its origin, and the latter one was reported to range between 40 and more than thousands of base pairs. In apoptosis, DNA is cleaved by endogenous endonucleases resulting in multiples of DNA fragments of 160–180 bp, corresponding to mononucleosomes (approx. 166 bp) and polynucleosomes [21]. This generates a ‘ladder pattern’ after electrophoretic separation, which can be observed in the case of cfDNA, suggesting apoptosis as one of its main origins. The presence of longer cfDNA fragments (larger than 10 kbp) is usually a result of the nonspecific degradation characterizing necrosis, while fragments of cfDNA <100 bp are enriched with mtDNA and circulating tumor DNA (ctDNA) [22]. Interestingly, apart from passive secretion mechanisms via various cell death pathways, DNA can also be released from living cells by active cellular secretion, although at present little is known about why functional cells secrete DNA and what is the biological significance of this process [23, 24].

## 3 Extracellular vesicles‐associated DNA

**Fig. 1:**
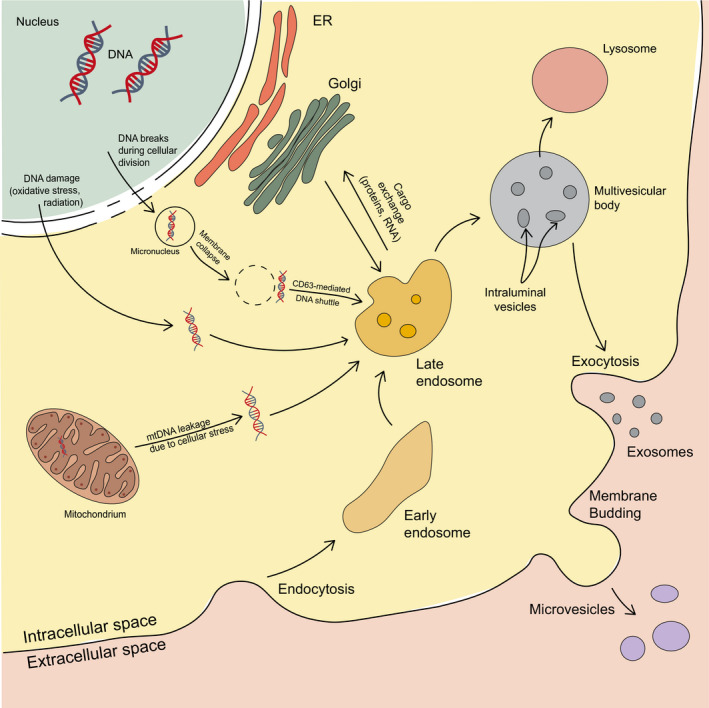
Extracellular vesicle biogenesis and possible mechanisms of DNA uptake. Microvesicles and exosomes are prevalent types of extracellular vesicles in biofluids. Microvesicles are generated via direct outward plasma membrane budding, during which components of cytoplasm can be incorporated into vesicles (including DNA). Exosomes biogenesis includes inward invagination of plasma membrane and formation of early endosomes, which subsequently mature into late endosomes and eventually form multivesicular bodies containing intraluminal vesicles (ultimately released as exosomes). Formation of intraluminal vesicles involves sequestering of molecular cargo including cytoplasmic DNA, both genomic and mitochondrial. DNA damage caused by cellular stress can lead to its leakage from nucleus or mitochondrium, exposing DNA to cytoplasm and facilitating its shuttle to intraluminal vesicles or integration into microvesicles.

One example of active release of DNA into the circulation is through EVs, heterogeneous membrane‐enclosed particles that play fundamental roles in cell‐to‐cell communication in both physiological and pathological processes [8, 25]. EVs can be classified according to their size and origin into three main categories: apoptotic bodies, microvesicles, and exosomes. Considering that the current isolation methods cannot provide pure preparations of exosomes or microvesicles, and no consensus has yet emerged on specific markers for subtyping EVs, current guidelines recommend to denominate these vesicles as EVs in general [26]. For the purposes of this review, except for instances where we will focus in specific aspects of EV biogenesis, we will follow this recommendation. It has been demonstrated that during their biogenesis, EVs associate with parental cell‐derived molecular cargo that include proteins, lipids, RNAs, as well as different types of DNA, including ssDNA, dsDNA, mtDNA, and even viral DNA [8, 27, 28, 29, 30, 31, 32] (Fig. 1). Interestingly, numerous studies have reported the presence of DNA attached both to the surface of EVs and in their lumen [32, 33, 34, 35].

Balaj *et al*. [36] examined the content of extracellular vesicles derived from cell culture as well as tumor‐bearing mice and showed that tumor‐derived vesicles contain ssDNA, both genomic (gDNA) and complementary DNA, and that this cargo reflects the genetic status of the tumor. The presence of DNA in tumor‐derived EVs was later confirmed by another study, in which the group investigated gDNA content in the subpopulations of EVs isolated from several prostate cancer cell lines, plasma of prostate cancer patients, and healthy subjects. Lazaro‐Ibanez *et al*. [37] demonstrated the presence of dsDNA in all analyzed EVs and showed that the gDNA content differed across subtypes of vesicles, suggesting a possible selective packing of DNA into various types of EVs.

### 3.1 DNA in microvesicles and apoptotic bodies

Microvesicles, ranging from 100 nm to 1 μm in size, are generated via direct outward budding of the plasma membrane of both living and dying cells, while the largest class of EVs, apoptotic bodies (1–5 μm), are formed during late phase of apoptotic programmed cell death and are the result of membrane blebbing [25]. This process leads to the release of vesicles containing substances from dying cells, including fragments of degraded DNA.

Vagner *et al*. [35] investigated the abundance of DNA in small and large EVs in plasma from prostate cancer patients and showed enrichment of gDNA in large EVs compared to smaller vesicles. Moreover, DNA in large vesicles contained the entire genome of parental cell, and tumor‐specific alterations could be detected in these vesicles.

### 3.2 DNA in exosomes

Exosomes differ from larger EVs because of their small size (ranging from 30 to 150 nm) and endocytic origin. The biogenesis of exosomes involves invagination of the plasma membrane and subsequent formation of early endosomes, which later mature into late endosomes. The latter ones undergo inward invagination and ultimately generate multivesicular bodies (MVBs) containing intraluminal vesicles (ILVs). ILVs are finally secreted as exosomes into the extracellular space upon MVB fusion with the plasma membrane [38]. Exosomes are released by virtually all cells, and even though they were considered as little more than cellular ‘trashbags’ until the 1990s, they have eventually emerged as crucial mediators in the tumor microenvironment, especially in the modulation of tumor immunity and angiogenesis, as well as growth and metastasis [39, 40, 41].

Different types of DNA have been reported to be associated with exosomes, including mtDNA and gDNA, although the relative abundance of different DNA cargo is still being investigated. Thakur *et al*. [32] were one of the first groups to demonstrate extraction of dsDNA from exosomes, using a panel of different cancer cell lines, and further showed that the analyzed exosomes contained DNA representing the entire genome and reflected the mutational status of the cell of origin. Later studies confirmed the presence of dsDNA fragments ranging from 100 bp to 20 kbp in size in exosomes and showed the ability of tumor‐derived exosomes to carry whole genomes of parental cells, supporting their potential application as sources of cancer biomarkers [29, 31]. Despite previous reports suggesting that the majority of circulating DNA is associated with larger EVs, Fernando *et al*. [27] analyzed DNA extracted directly from whole human blood plasma and from plasma exosomes, and demonstrated that more than 90% of cell‐free DNA is associated with exosomes.

At present, very little is known about the origins of exosomal DNA and the presence of nuclear content in exosomes raises a debate on how gDNA located in the nucleus could be packaged into these vesicles. One of the potential mechanisms for DNA to be loaded into exosomes involves their biogenesis, when early endosomes mature into late endosomes and accumulate ILVs. The formation of ILVs includes the sequestering of proteins, lipids, and cytosol, which could lead to the encapsulation of cytosolic DNA within the vesicles, although the relation between cytosolic levels of DNA and EV‐DNA has not been established yet. A more detailed mechanism was proposed by Yokoi *et al*. and involves micronuclei (MN), nuclear membrane‐enclosed structures that originate from chromatid fragments caused by misrepaired DNA breaks and unrepaired DNA breaks, from malsegregation of chromosomes, and other malsegregation/misrepair of DNA [42, 43, 44]. Due to the instability of their nuclear membrane, micronuclei were shown to occasionally collapse, exposing their nuclear content to the cytoplasm. In the cytoplasm, gDNA could be shuttled to MVBs and ultimately loaded into exosomes. In fact, increased packaging of DNA in EVs was described in genotoxic conditions and cancer [44], where MN formation is increased. Yokoi *et al*. also observed that cancer cell treatment with genotoxic drugs increased MN production and resulted in higher exosome release. It has been proposed that gDNA segregated in MN can be transcriptionally active or inactive, depending on whether MN would contain whole chromosomes or acentric fragments of DNA. Alternatively, MN DNA can be degraded through apoptotic‐like mechanisms, or expulsed from the cell [45], potentially via DNA packaging into EVs. The consequence of DNA expulsion can potentially be the restoration of normal cellular status, when extra chromosomes and/or copies of genes are expulsed, or loss of gene dosage, when MN content is complementary to the main nucleus [45]. Moreover, Yokoi *et al*. demonstrated that micronuclei and exosomes can actively interact in live cells and share their content, including nuclear proteins. The authors analyzed the tetraspanin CD63, which is involved in loading exosomal cargo, and revealed that it surrounds the MN envelope and could have a role in shuttling nuclear content to exosomes by forming complexes with gDNA via nuclear proteins.

At some level challenging all presented results, Jeppesen *et al*. [46] reassessed exosome composition and reported that dsDNA, as well as DNA‐binding histones, is not being carried by exosomes and suggested an exosome‐independent mechanism of DNA secretion via amphisomes. However, this study had several limitations, including unreported amount of exosomes used in the study, as well as a limited number of cell lines included in the report, which are both important aspects that need to be taken into consideration while analyzing DNA presence in EVs.

## 4 Biological significance of EV‐DNA

The packaging of DNA within the lumen of membrane‐enclosed vesicles confers increased stability to it by protecting it from the external environment and preventing recognition by the immune system [47]. However, the biological function of EV‐DNA is still largely unknown, despite a number of research works supporting the concept that EV‐DNA may have an important physiological impact on the cells of origin as well as recipient cells, by playing a role in maintenance of cellular homeostasis and acting as an intercellular messenger.

### 4.1 Cellular homeostasis

It is well established that the presence of DNA in the cytoplasm is related to cellular damage, tumorigenesis, or infection, and leads to the activation of DNA‐sensing pathways [48]. Takahashi *et al*. proposed that EVs contribute to the maintenance of cellular homeostasis by excreting damaged DNA from cells and preventing an aberrant activation of the innate immune response. The inhibition of exosome release resulted in the accumulation of harmful nuclear DNA in the cytoplasm of these cells, suggesting that damaged DNA may be one of the sources of EV‐DNA [49]. This result was further supported by Torralba *et al*. [50], who demonstrated the presence of partially oxidized mtDNA in EVs. As many mechanisms of DNA release from cells are a consequence of various cell death pathways, including apoptotic bodies, EV‐DNA could be regarded by some as a ‘degradation product’ from dying cells. Interestingly, studies show that the release of EV‐DNA seems to be associated with cell survival rather than cell death. Takahashi *et al*. reported that there is in fact an inverse correlation of EV release with apoptosis, suggesting that the release of EV‐DNA is not an indicator of cell death. Moreover, Wang *et al*. [51] showed that the concentration of circulating DNA correlated with the percent of cells in G1 phase and suggested the active release of DNA via EVs.

### 4.2 DNA transfer and modulation of recipient cells

The potential functionality of circulating DNA has gained increased interest with reports showing its ability to integrate into the genomes of recipient cells, thus suggesting that it could transform them [52, 53]. These results raised the question of whether DNA associated with vesicles could also be taken up by recipient cells and what would be the effect on these cells. In 1999, Holmgren *et al*. demonstrated that the DNA cargo of apoptotic bodies may be involved in horizontal gene transfer: they showed that engulfment of lymphoma‐derived apoptotic bodies by fibroblasts resulted in the incorporation of DNA from these vesicles into the genome of fibroblasts [54]. EV‐DNA‐mediated transfer of genes was later confirmed by incubation of mesenchymal stromal cells with EVs transduced with *Arabidopsis thaliana* DNA, which resulted in a stable integration of this DNA into the genome of the recipient cells [33].

Extracellular vesicle‐mediated horizontal transfer of DNA and its modulating ability was further explored by Cai *et al*. [55], who showed that EV‐DNA can not only be transferred to recipient cells, but also cause an increase in gDNA‐coding mRNA as well as protein expression. The group suggested that EV‐DNA may have a physiological influence on the recipient cells, as they demonstrated EV‐DNA‐mediated transfer of a BCR/ABL hybrid gene, involved in chronic myeloid leukemia, to neutrophils and HEK293 cells. In a follow‐up study, the same group confirmed their previous results in an *in vivo* model, showing that EV transfer of BCR/ABL gene causes chronic myeloid leukemia in immunodeficient mice [56]. In 2014, Lee *et al*. [57] reported the transfer of full‐length dsDNA fragments of oncogenic H‐*ras* via EVs and their incorporation into the genome of recipient nontransformed epithelial cells resulted in increased proliferation. However, in their more recent work, the group showed that these alterations are not permanent due to the barrier mechanisms that diminish uptake, retention, and function of oncogenic H‐*ras*, thus raising a debate on the preexisting cellular conditions that may influence the EV‐mediated transfer and incorporation of oncogenes or other exogenous DNA [31].

Interesting results have been also presented regarding transfer and function of other types of EV‐DNA, including mtDNA. Guescini *et al*. [28] demonstrated for the first time the existence of mtDNA in EVs and further showed that it could be delivered to other cells, proposing that this could be a mechanism for the transference of altered mtDNA in various pathologies, including Alzheimer's disease. The implication of EV‐mtDNA transfer in the development of therapy resistance was shown in breast cancer. Horizontal transfer of EV‐mtDNA derived from cancer‐associated fibroblasts to therapy‐induced dormant breast cancer cells promoted escape from dormancy of these cells and led to hormone therapy resistance [58].

### 4.3 Regulation of immune response

EV‐DNA plays a role in inflammatory responses, which suggests its involvement in inflammation‐related states such as aging, and autoimmune and neurodegenerative diseases [59, 60]. In fact, the presence of cfDNA in the circulation induces different types of inflammatory reactions, including toll‐like receptors‐mediated immune stimulation and other nucleic acid‐sensing mechanisms [61]. One of these sensors, cyclic GMP‐AMP synthase (cGAS)‐stimulator of interferon genes (STING), is a cytosolic DNA‐sensing pathway that triggers the expression of inflammatory genes and leads to the induction of interferon type 1 (IFN1) response. Interestingly, EV‐DNA is also capable of eliciting and regulating immune responses, including the cGAS‐STING pathway. The immunostimulatory effect of EV‐DNA was shown by Kitai *et al*. [62], where treatment of breast cancer cells with an antitumor agent prompted secretion of DNA‐containing EVs and subsequent activation of dendritic cells via the cGAS‐STING pathway, eliciting antitumor immunity. Moreover, upon antigen stimulation, T cells can secrete EVs containing both gDNA and mtDNA. This triggers an IFN1 response in dendritic cells also via the cGAS‐STING pathway, priming them for subsequent infections [50].

The role of EVs in the innate immune response was also described in the case of virus infections, where Kouwaki *et al*. [30] showed that cells infected with hepatitis B virus (HBV) release EVs containing viral dsDNA that regulate the antiviral response by stimulating macrophages. Similarly, EVs produced by Kaposi's sarcoma‐associated herpesvirus‐infected cells induce interferon‐stimulated genes by the presence of mtDNA on their surface, supporting the function of EV‐DNA in stimulation of the antiviral immune response [63]. In models of partial hepatectomy, it was found that the procedure was followed by the release of EVs with abundant nucleic acid cargo, which may serve as a ligand for activation of mitochondrial antiviral signaling protein and STING pathways. Since the deficiency of mitochondrial antiviral signaling protein and STING delays liver regeneration after partial hepatectomy, the immunostimulatory effect of EV‐DNA could contribute to liver regeneration [64]. In another study, mtDNA‐containing EVs were found to be capable of inducing an inflammatory response in nonoxidatively stressed epithelial cells *in vitro*. Administration of isolated mtDNA into the lungs of naïve mice induced the production of pro‐inflammatory mediators, without histopathologic evidence of tissue injury; thus, mtDNA‐specific damage seems to be a mechanism that links prolonged, low‐level oxidative stress to autocrine and paracrine inflammation during the early stages of inflammatory lung disease [65].

## 5 Technologies for EV‐DNA characterization and analysis

As the presence of DNA in EVs gained attention in terms of its potential clinical value as a biomarker, development and optimization of protocols for DNA extraction and analysis became critical. Strategies for EV‐DNA purification and characterization are still not well established, and, since the methods may affect the purity and yield of DNA, their pros and cons need to be considered depending on the downstream application. At present, the most commonly applied techniques for quantity and quality assessment of vesicular DNA include Qubit fluorometer and NanoDrop measurements, and capillary electrophoresis‐based technologies like Bioanalyzer of Fragment Analyzer. Regarding EV‐DNA analysis, the most commonly used approaches comprise standard methods for DNA sequence and mutation analysis, including different variants of polymerase chain reaction (PCR) as well as next‐generation sequencing (NGS) [27, 29, 32].

Over the last few years, flow cytometric analysis of EVs has been explored, but standards for labeling EV‐DNA for flow cytometry have not yet been established. A few strategies have been described in the literature, using DNA dyes such as propidium iodide, Pico Green, and DRAQ5 that have previously been used in cell cycle or live/dead analysis. The results of these studies are promising as they confirm the heterogeneity of the EV‐DNA load and illustrate that this load indeed changes in response to antibiotics, genotoxic agents, and molecular targeted therapy [e.g., with epidermal growth factor receptor (EGFR) inhibitors] [34, 43, 66].

## 6 Applications of EV‐DNA in the clinical setting

As the presence and significance of EV‐DNA in pathophysiologic processes became more evident, it became inevitable that EV‐DNA began to be tested in clinical studies, especially in the areas of cancer, infectious diseases, and pregnancy and prenatal diagnosis. An overview of these studies is summarized in Table 1.

**Table 1:** EV‐DNA clinical studies

References	Clinical condition	Summary
Cancer			
Choi *et al*. [66], Montermini *et al*. [69]	Lung and epidermal cancer	EGFR inhibitors increased emission of EVs harboring EGFR and gDNA *in vitro*
Yokoi *et al*. [43]	Ovarian cancer	Treatment with olaparib and topotecan increased DNA in ovarian cancer cell exosomes
Yang *et al*. [71], Allenson *et al*. [72], Bernard *et al*. [73]	PDAC	KRASG12D and TP53R273H mutations can distinguish healthy controls and patients with pancreas‐associated pathologies from PDAC patients. KRAS mutations can differentiate between stages of disease and are associated with disease‐free survival after resection. EV‐DNA level after neoadjuvant therapy can predict response; MAF is associated with disease progression and a predictor of progression‐free and overall survival
Garcia‐Romero *et al*. [75]	Glioma	DNA‐containing EVs can cross the BBB and can be isolated from peripheral blood
Mata‐Rocha *et al*. [74]	Cervical cancer	EV‐DNA, HPV E6, and E7 oncogenes can be detected in pap smear samples
Lee *et al*. [76]	Bladder carcinoma	Bladder cancer‐associated mutations amplified in urinary EV‐DNA
Cancer treatment adverse effects			
Lian *et al*. [78]	Irinotecan‐induced diarrhea	DNA‐containing EVs from irinotecan‐treated cells can launch inflammation pathways in immune cells through AIM2
Ariyoshi *et al*. [79]	Radiation‐induced bystander effect	EVs carrying mtDNA induced DNA damage in human dermal fibroblast cells
Infections			
Sukriti *et al*. [82] Sanada *et al*. [81]	Hepatitis B	EVs isolated from CHB carriers carry HBV DNA and transmit HBV DNA into noninfected cells
Morris‐Love *et al*. [83]	Progressive multifocal leukoencephalopathy	JC polyomavirus can be released from and transmitted to glia by EVs
De Carolis *et al*. [84]	HPV infection	EV HPV DNA is a trigger for breast cancer niche aggressiveness
Cho *et al*. [85]	Tuberculosis	EV‐DNA can detect *M. tuberculosis* DNA in respiratory samples
Pregnancy and prenatal diagnosis			
Orozco *et al*. [88]	Preeclampsia	Term preeclamptic women had higher levels of EV‐DNA than control pregnant women

### 6.1 Cancer

Liquid biopsies have been studied in cancer more than in any other disease entity. This reflects the relatively high burden of this disease in society [67], and the fact that liquid biopsies enable constant monitoring of a reliable biomarker, which is the optimal method to identify response to treatment [68].

#### 6.1.1 EV‐DNA‐based liquid biopsies in specific cancer types

Choi *et al*. [66] studied the effects of the anti‐oncogenic EGFR inhibitors dacomitinib and canertinib on EV‐DNA cargo in lung cancer cells and found that treatment with these agents resulted in increased release of EVs harboring both EGFR and gDNA. At baseline, both control and inhibitor‐treated A431 lung cancer cells released DNA‐positive EVs of different sizes; however, the total number of DNA‐carrying EVs increased following exposure to the EGFR inhibitors, and also led to the emergence of another population of DNA‐positive nonvesicular particles. Pretreatment with a caspase inhibitor prevented the increased release of DNA‐positive particles in response to EGFR inhibitors, suggesting involvement of apoptotic vesiculation pathways in this process [66]. A similar study involving EGFR mutation in epidermoid carcinoma showed that treatment of EGFR‐driven tumor cells with canertinib and dacomitinib, but not with anti‐EGFR antibody (cetuximab) or etoposide, increased the release of exosome‐like EVs containing EGFR and gDNA. The EGFR/gDNA co‐expression in exosomes signifies effective antitumor EGFR kinase inhibitor treatment, as this coincided with a dramatic reduction in cellular phosphorylated EGFR levels and diminished cell viability [69]. This is an important aspect of EV‐DNA studies, as the co‐expression of circulating DNA with another molecular target cannot be studied using cfDNA alone [66, 70].

A similar shift in DNA in post‐treatment EVs was described in a study on ovarian cancer by Yokoi *et al*. Using imaging flow cytometry, they demonstrated that 10% of EVs derived from cancer cells and < 1% of EVs derived from blood and ascites from patients with ovarian cancer carry nuclear contents. They also found that treatment with the genotoxic drugs olaparib and topotecan resulted in increased DNA in ovarian cancer cell EVs, both *in vitro* and *in vivo* [43].

A proof‐of‐concept study by Yang *et al*. regarding the potential clinical utility of circulating EV‐DNA for identification of KRASG12D and TP53R273H mutations in pancreatic ductal adenocarcinoma (PDAC) patients showed that digital PCR analysis of EV‐DNA can potentially differentiate healthy controls from cancer patients. In addition, this method has also been able to differentiate patients with malignancy from those who had pancreas‐associated pathologies, including chronic pancreatitis and intraductal papillary mucinous neoplasm. This study highlights the value of circulating EV‐DNA for a rapid, low‐cost identification of cancer‐driving mutations. However, the authors caution that despite the good sensitivity, the specificity of the test is still not validated for the detection of cancer in clinical settings [71]. Studies by Allenson *et al*. and Bernard *et al*. showed that EV‐DNA‐detected KRAS mutations can be used to differentiate between patients at different stages of the disease, as well as from healthy controls. Mutant KRAS EV‐DNA was detected in 43.6% of early‐stage PDAC patients, compared to 66.7%, 80%, and 85% of localized, locally advanced, and metastatic PDAC patients, respectively. While the KRAS mutation detection rate for localized and metastatic pancreatic cancer was nearly equivalent for cfDNA and EV‐DNA, concordance to surgically resected primary tissue samples was 95.5% for EV‐DNA and only 68.2% for cfDNA. Likewise, tissue versus liquid biopsy concordance from 12 samples derived by fine‐needle aspirates was 83.3% for EV‐DNA and only 66.8% for cfDNA [72, 73]. It was also found that an individual with a positive EV‐DNA KRAS mutant status was eight times more likely to have early‐stage PDAC than to be cancer‐free. In addition, it was observed that mean frequencies of the KRAS mutation allele (MAFs) have the same pattern, as MAFs were higher in metastatic compared with localized disease (mean of 10.09% versus 2.7%). Mean KRAS MAFs were also associated with disease‐free survival after resection (median disease‐free survival of 441 versus 127 days for patients with less than 1% MAF compared with those with more than 1% MAF). The same group further confirmed the clinical relevance of EV‐DNA in PDAC by comparing EV‐DNA with ctDNA. In 34 patients with potentially resectable tumors, an increase in EV‐DNA level after neoadjuvant therapy was significantly associated with disease progression, whereas ctDNA levels did not show a correlation with the outcome. However, a different MAF cutoff compared to the previous study (MAFs ≥ 5%) in EV‐DNA was found to be a significant predictor of progression‐free survival and overall survival, and a MAF peak above 1% in EV‐DNA was significantly associated with radiologic progression [72, 73]. Collectively, these studies indicate that the use of EV‐DNA to identify KRAS mutations for early detection and disease prognosis is feasible; however, bigger cohorts may be needed in order to validate this test for clinical use.

In cervical cancer, another valuable application was the detection of cancer‐causing human papillomavirus (HPV) DNA from pap smear samples of patients with no visible or with low‐grade cervical lesions. In these cases, the HPV DNA is in episomal form and not yet integrated into the host genome. Using NGS followed by confirmatory PCR, Mata‐Rocha *et al*. were able to confirm the presence of the HPV E6 and E7 oncogenes in EVs from both HeLA cells conditioned media and the patient‐derived cervical samples. This confirms that EVs can be a source of DNA for the detection of an HPV infection, regardless of whether viral DNA is integrated or episomal, and suggests the possibility of HPV transmission between cells through EVs [74].

#### 6.1.2 Detection of EV‐DNA in various biofluids

A valuable advantage of EV‐associated liquid biopsies is the ubiquitous presence of EVs in biofluids. They can cross vascular barriers, such as an intact blood–brain barrier (BBB), and be detected in the peripheral circulation [75]. This provides a minimally invasive method for the detection of central nervous system tumors compared to previous methods such as sampling the cerebrospinal fluid or an outright tumor biopsy. Garcia‐Romero *et al*. used an orthotopic xenotransplant model of human cancer stem cells which produced a disseminated brain tumor phenotype featuring an intact BBB. They found that the EVs isolated and enriched from the peripheral blood carried human gDNA sequences corresponding to those of the xenotransplanted cells. In a cohort of glioma patients with an intact BBB, the authors were also able to demonstrate that peripheral blood EV cargo can be successfully used to detect the presence of specific mutations, such as IDH1G395A. This is clinically relevant, as IDH1‐mutated patients are expected to have longer survival rates than their counterparts with non‐IDH1‐mutated tumors, despite similar histopathologic features [75]. It is also worth noting that other body fluids, such as urine, can provide viable material for EV‐DNA‐based liquid biopsies. Lee *et al*. analyzed whether genetic alteration in urothelial bladder carcinoma was reflected in urinary cfDNA or EV‐DNA, and found a concordance between the copy number profiles of tumor tissue and urinary DNA (cfDNA and EV‐DNA) with allelic frequencies of 56.2% and 65.6%, respectively. Amplification of MDM2, ERBB2, CCND1, and CCNE1 and deletion of CDKN2A, PTEN, and RB1, all known to be frequently altered in bladder carcinoma, were also identified. Also, copy number variation plots of cfDNA and EV‐DNA showed a similar pattern to those from the tumor samples [76].

#### 6.1.3 Role of EV‐DNA in treatment‐related adverse effects

The study of EV‐DNA in cancer has not only shown benefits in terms of determining the status of disease, but also in evaluating the occurrence of adverse side effects such as chemotherapy‐induced diarrhea and radiation‐induced bystander effect. Lian *et al*. explored the immune‐mediated mechanisms of irinotecan‐induced diarrhea in colorectal cancer and found that double‐stranded DNA in EVs can launch inflammation pathways in immune cells through the cytosolic DNA sensor AIM2. They also discovered that a majority of dsDNA contained in the cell culture supernatant of irinotecan‐treated cells was associated with EVs, and the authors were able to establish a connection between exosome release and irinotecan‐induced inflammation *in vivo* in irinotecan‐treated mice [77, 78]. In addition, DNA‐damaging anticancer therapy—such as cisplatin, etoposide, or radiation therapy—has been shown to trigger innate immune responses that involve the leakage of nuclear‐derived self‐DNA from tumor cells. EV‐DNA from these states has been found to be either nuclear gDNA or mtDNA [78]. In terms of radiation therapy, released DNA‐containing EVs have been found to enter adjacent cells as a result of exosome migration and internalization and thus prompt radiation‐induced bystander effect—a phenomenon in which some tissues, despite not being directly in the radiation treatment field, exhibit radiation‐induced effects like DNA damage and necrosis. Specifically, secretion of mtDNA through EVs has also been found to be involved in mediating radiation‐induced bystander effect signals [79, 80].

### 6.2 Infections

Infectious processes also seem to involve EV‐DNA. For example, purified EVs from HBV‐infected humanized chimeric mouse serum were found to contain HBV DNA and were capable of transmitting it to naive primary human hepatocytes [81, 82]. Another example is a study by Morris‐Love *et al*., who revealed that human polyomavirus 2, a major cause of progressive multifocal leukoencephalopathy in immunocompromised patients, can be released from and transmitted to other glial cells by EVs. By being protected from neutralizing antibodies, this mode of transmission leads to increased virulence [83]. In a similar fashion, De Carolis *et al*. showed that HPV DNA can be found in EVs derived from breast cancer patients' sera. Interestingly, they also showed that HPV DNA can be transferred by EVs to recipient breast cancer stromal cells, suggesting that EV HPV DNA is a potential trigger for breast cancer niche aggressiveness [84]. A practical diagnostic application of EV‐DNA for infectious diseases was demonstrated by Cho *et al*., who utilized total DNA and EV‐DNA on a droplet digital PCR platform to detect *Mycobacterium tuberculosis* DNA in respiratory samples of patients with suspected pulmonary infection. Compared with mycobacterial culture, sensitivity and specificity of droplet digital PCR were 61.5% and 98.0% using total DNA, and 76.9% and 98.0% using EV‐DNA, respectively [85]. This suggests that the utilization of EV‐DNA can result in more sensitive and accurate diagnostic tests, which is particularly important for immediate and effective patient treatment and infection control.

### 6.3 Pregnancy and prenatal diagnosis

During pregnancy, immunomodulation may result from EVs released from the placental trophoblast into the maternal circulation. EVs contribute to the amount of total fetal cfDNA, which in turn plays a role in pregnancy complications and fetal rejection, and possibly spontaneous abortion and preterm delivery as well [86, 87]. There is increasing evidence that EVs play an important role in the pathogenesis of systemic lupus erythematosus and rheumatoid arthritis in pregnant women. In addition, these EVs carry proteins and DNA that could mediate pro‐inflammatory effects or serve as potential markers of autoimmune diseases [86]. It was also reported that cell‐free nucleic acids are present in the mother's circulation, either in the form of apoptotic bodies or as syncytiotrophoblast debris, and are increased during fetal growth restriction as well as preeclampsia [88].

### 6.4 Clinical trials testing EV‐DNA

Despite more than a hundred active clinical trials (as registered in ClinicalTrials.gov) testing EVs, only a handful of them focus on the study of EV‐DNA. Some of these studies aim to identify biomarkers following experimental treatment. These include NCT03217266, a phase 1b trial which investigates the use of MDM2 inhibitor AMG‐232 (KRT‐232) and radiation therapy in patients with soft tissue sarcoma, with a secondary objective of measuring tumor mutations in ctDNA as well as EV‐DNA and RNA [89]. Another example is NCT03228277, a single‐arm, open‐label, completed phase 2 study to assess the efficacy of olmutinib (as measured by objective response rate) in T790M‐positive non‐small‐cell lung cancer (NSCLC) patients confirmed using EV‐DNA obtained from bronchoalveolar lavage fluid [90]. Others intend to confirm the technical feasibility of detecting mutations in EV‐DNA, like NCT03236675, which aims to demonstrate detection of EML4‐ALK fusion transcripts and T790M EGFR mutation from EVs in the circulation of NSCLC patients [91]. This underscores that the study of EV‐DNA in the clinical context is still quite open for further validation.

## 7 Current challenges and conclusions

Overall, EV‐DNA as a biomaterial for liquid biopsies is a new but definitely promising area of study. The evidence presented in this review demonstrates that mutations detected in EV‐DNA can indeed differentiate between healthy and diseased states. EV‐DNA cargo changes in response to treatment, and this uncovers the link between cellular response and tissue environment inflammation, which may be relevant in immunotherapy, autoimmune diseases, and others. In spite of the encouraging progress in the use of EV‐DNA as a source of disease biomarkers, current protocols cannot provide definitive solutions to identify and isolate EVs from specific sources (e.g., tumor cells). Therefore, one of the pitfalls of utilizing EVs, and essentially any liquid biopsy component that includes circulating tumor DNA as a surrogate for the tumor genome, is that genetic information obtained from these components will be diluted in large part with the DNA contained on EVs derived from noncancerous cells. However, the discovery of surface biomarkers that identify EVs derived from specific cell types has the potential to provide a strategy to separate specific populations of EVs and enrich isolates with DNA from the cell populations of interest [70, 92]. We look forward to improved strategies to study EV‐DNA and the establishment of more refined ways of using this in liquid biopsies as a standard of care.

## Conflict of interest

The authors declare no conflict of interest.
